# Genetic Influences on the Development of Fibrosis in Crohn’s Disease

**DOI:** 10.3389/fmed.2016.00024

**Published:** 2016-05-30

**Authors:** Bram Verstockt, Isabelle Cleynen

**Affiliations:** ^1^Department of Medicine and Cambridge Institute for Medical Research, University of Cambridge School of Clinical Medicine, Cambridge, UK; ^2^Translational Research in Gastrointestinal Disorders (TARGID), Department of Clinical and Experimental Medicine, KU Leuven, Leuven, Belgium; ^3^Laboratory of Complex Genetics, Department of Human Genetics, KU Leuven, Leuven, Belgium

**Keywords:** stricturing disease, fibrosis, Crohn’s disease, genetics, NOD2

## Abstract

Fibrostenotic strictures are an important complication in patients with Crohn’s disease (CD), very often necessitating surgery. This fibrotic process develops in a genetically susceptible individual and is influenced by an interplay with environmental, immunological, and disease-related factors. A deeper understanding of the genetic factors driving this fibrostenotic process might help to unravel the pathogenesis, and ultimately lead to development of new, anti-fibrotic therapy. Here, we review the genetic factors that have been associated with the development of fibrosis in patients with CD, as well as their potential pathophysiological mechanism(s). We also hypothesize on clinical implications, if any, and future research directions.

## Introduction

Crohn’s disease (CD) is a chronic, inflammatory disease of the gastrointestinal tract, which predominantly affects the distal ileum and right colon. Although many patients express an inflammatory phenotype at diagnosis, the natural evolution of CD is commonly a progression toward a fibrostenotic or penetrating disease in 60% of patients (1, 2). The pathogenesis of CD is multifactorial, including a genetic background (3). Genome-wide association studies and meta-analysis have identified a total of 200 inflammatory bowel disease (IBD) risk loci thus far (4, 5).

Transmural inflammation as seen in CD can cause irreversible changes in the intestinal architecture, over time leading to fibrotic strictures necessitating surgery in 30–50% of patients (1, 2, 6). Recurrence of these fibrotic lesions occurs in 23–41% of CD patients, requiring additional resections (7), which in turn reinforce the recurrence rate (8). These fibrostenotic strictures are the result of a failure of physiological wound healing. The activation of mesenchymal cells by cytokines, growth factors, and other mediators released by immune cells, epithelial cells, and mesenchymal cells themselves are believed to play an important role (9–11). The development of fibrosis in CD is influenced by various genetic, environmental, immunological, and disease-related factors (12–15). So far, the relative contribution of each component in the pathogenesis is not clear. This review aims to clarify the genetic contribution in developing fibrosis in patients with CD.

## Genetics and Fibrosis

There exists broad heterogeneity of published literature concerning the genetic background of fibrotic CD. Below, we give an overview of individual variants and genes that have been associated with fibrotic disease in CD (Table 1), and we hypothesize on the potential pathophysiological mechanisms.

**Table 1:** **Key gene polymorphisms and their significance in intestinal fibrosis in CD**.

Gene	Polymorphism	Association	Studied population	Sample size^e^	Reference
*NOD2*	rs2066844, R702W	Discussed separately in Table 2			
	rs2066845, G908R				
	rs2066847, Leu1007fsinC				
*ATG16L1*	rs2241880, T300A	Ileal disease location	Australian	669–154	Fowler et al. (58)
		Fibrostenotic disease^a^			
*IL-23R*	rs1004819	Ileal disease location^b^	German	833	Glas et al. (65)
		Fibrostenotic disease^a^^,^^b^			
*CX3CR1*	rs3732379, V249I/rs3732378, T280M	Ileocolonic disease location	German	206	Brand et al. (74)
		Fibrostenotic disease^a^			
	rs3732379, V249I	Fibrostenotic disease^a^	Caucasian	239	Sabate et al. (52)
*TGF*-β	rs1800471, R25P	Fibrostenotic disease^c^	Australian	235–112	Hume et al. (79)
*MMP-3*	−1613 5T6T	Colonic disease location	Dutch	134	Meijer et al. (88)
		Fibrostenotic disease^a^			
*MAGI1*	rs11924265	Fibrostenotic disease^d^	Spanish	1090–1296	Alonso et al. (96)
*JAK2*	rs10758669	Ileal disease location	Caucasian	1528	Cleynen et al. (50)
		Fibrostenotic disease^d^			
*FUT2*	rs601338	Fibrostenotic disease^d^	Belgian	647	Forni et al. (99)
*IL12B*	rs1363670	Fibrostenotic disease^d^	Belgian	875	Henckaerts et al. (103)

*If a significant association between the given variant and disease location is found in the reference, this is mentioned in the table*.

*^a^Not corrected for disease location*.

*^b^Not significant after Bonferroni correction*.

*^c^No longer significant after multivariate analysis taking into account disease location*.

*^d^Corrected for disease location*.

*^e^Number of included CD patients in primary cohort − number of included CD patients in replication cohort (if applicable)*.

### Nucleotide-Binding Oligomerization Domain-Containing Protein 2

The *NOD2* gene is the most studied gene in relation to fibrostenotic disease in CD. Located in the IBD1 locus on chromosome 16q12, it encodes CARD15, a member of the Apaf-1/NOD1 family of CARD (caspase recruitment domain-containing protein) proteins (16, 17). NOD2/CARD15 is mainly expressed by monocytes and macrophages, where it acts as a cytosolic sensor for bacterial products, and is involved in apoptosis and activates NF-κB in response to lipopolysaccharide (LPS) binding at its leucine-rich repeating region (LRR) (18, 19). Moreover, the CARD-domain provides CARD15 the unique function to be able to induce interleukin 1-beta (IL-1β) processing and release (20). Importantly, *NOD2* is also expressed in Paneth cells (21).

In the early 2000s, three *NOD2* variants, including two amino acid substitutions (R702W in exon 4 and G908R in exon 8) and one frameshift mutation (Leu1007fsinC in exon 11), were identified as associated with CD (17, 22–25). Later on, several other *NOD2* SNPs were found to be associated with CD, although the first three described still represent the strongest association signals. Ever since *NOD2* was identified as a CD susceptibility gene, many genotype–phenotype studies were performed to find its role in defining CD disease location and behavior, but none of the three SNPs was uniformly found as an independent risk factor for developing fibrostenotic disease in CD (12, 15, 18, 19, 21, 24, 26–53). Some genotype–phenotype studies demonstrated strong associations between at least one of the three *NOD2* variants and fibrostenotic disease (19, 32, 38, 52), often independent of an association with small bowel disease (18, 26, 31, 37, 50) (Table 2).

**Table 2:** **Overview of original studies showing an association between NOD2 and fibrotic CD**.

Polymorphism	Association	Studied population	Sample size	Reference
rs2066844	Fibrostenotic disease^a^	French	205	Heresbach et al. (34)
R702W				
rs2066845	Fibrostenotic disease^c^	Spanish	204	Mendoza et al. (33)
G908R	Fibrostenotic disease^b^	Meta-analysis	8833	Adler et al. (47)
rs2066847	Ileal disease location	North-American	201	Abreu et al. (18)
Leu1007fsinC	Fibrostenotic disease^b^			
	Ileal disease location	Italian	133	Vavassori et al. (29)
	Fibrostenotic disease^c^			
	Fibrostenotic disease^c^	German	97	Radlmayer et al. (28)
	Ileal disease location	Italian	316	Annese et al. (37)
	Fibrostenotic disease^b^			
	Fibrostenotic disease^c^	German	80	Seiderer et al. (53)
	Ileal disease location	German	303	Seiderer et al. (24)
	Fibrostenotic disease^c^			
	Ileal disease location	Caucasian	1528	Cleynen et al. (50)
	Fibrostenotic disease^a^			
	Ileal disease location	German	550	Schnitzler et al. (51)
	Fibrostenotic disease^c^			
All SNPs combined	Ileal disease location	British	244	Ahmad et al. (19)
	Fibrostenotic disease^d^			
	Ileal disease location	Finnish	271	Heliö et al. (32)
	Fibrostenotic disease^c^			
	Ileal disease location	Hungarian	527	Lakatos et al. (38)
	Fibrostenotic disease^c^			
	Ileal disease location	North-American	201	Abreu et al. (18)
	Fibrostenotic disease^a^			
	Colonic disease location	Caucasian	453	Lesage et al. (26)
	Fibrostenotic disease^a^			
	Ileal disease location	North-American	275	Brant et al. (31)
	Fibrostenotic disease^a^			
	Ileal disease location	Italian	316	Annese et al. (37)
	Fibrostenotic disease^a^			
	Ileal disease location	Caucasian	1528	Cleynen et al. (50)
	Fibrostenotic disease^a^			
	Ileal disease location	Spanish	239	Sabate et al. (52)
	Fibrostenotic disease^c^			

*If a significant association between the given variant and disease location is found in the reference, this is mentioned in the table*.

*^a^Corrected for disease location*.

*^b^Unclear if corrected for disease location*.

*^c^Not corrected for disease location*.

*^d^No longer significant after multivariate analysis taking into account disease location*.

The lack of uniformity concerning this topic seems mainly based on the small sub-analyses done in the different studies (Table 2). In 2004, Heresbach et al. observed in a Northern-French population *NOD2* R702W (rs2066844) as being a strong predictor of fibrostenotic disease, independently of ileal localization of the disease (34). No other group could confirm this association. An association of *NOD2* G908R (rs2066845) and fibrostenotic disease was first reported in a Spanish CD cohort, although fibrostenotic disease was mainly dependent on location of disease in the terminal ileum (33). Later on, Adler et al. reported in their meta-analysis G908R as being associated with fibrostenotic disease [pooled relative risk (RR) = 1.90] (47). It is important to highlight that only 12 of all included studies in this meta-analysis had enough data to analyze individual *NOD2* variants, and most included studies did not differentiate between G908R homo- and heterozygotes. Of the three *NOD2* variants, the Leu1007fsinsC frameshift mutation (rs2066847) shows the strongest association with fibrostenotic disease (18, 37). The same finding was also published by Radlmayr et al., who moreover reported no association with ileal disease (28). Vavassori et al. also noticed an association between Leu1007fsinC and fibrostenotic disease, although no correction for ileal disease involvement was made (29). Seiderer et al. calculated a positive predictive value (PPV) of 80% and a negative predictive value (NPV) of 75% for the diagnosis of small bowel stenosis in clinically symptomatic patients with a Leu1007fsinC variant. Furthermore, they noticed 62% of their patients being Leu1007fsinC homo- or heterozygous needed surgery, whereas the need for surgical intervention in patients without this variant was remarkably low (53). A sub-analysis of another cohort with 19 patients, all Leu1007fsinC homozygous, identified a high-risk population, characterized by, for instance, long-segment stenosis, frequent need for surgery, and high risk for re-stenosis afterward (24). The same group confirmed these findings later on in a prospective study (53), whereafter the European IBD chip project reported the same in a retrospective study (*n* = 38) (50), as did Schnitzler et al. (51). Besides studying the association of individual *NOD2* SNPs with a fibrostenotic CD phenotype, often the *NOD2* SNPs are considered together. The pooled RR of stricturing disease with the presence of any *NOD2* variant allele was 1.33 in a meta-analysis, including 35 studies by Adler et al. (47). Furthermore, Lesage et al. clearly described the “gene dosage effect” of *NOD2* SNPs: patients carrying two SNPs have a higher incidence of stenosis compared to patients with one or two wild-type alleles (26), which was afterward confirmed by others (31, 40, 47). Although many groups, thus, reported an association between *NOD2* variants and fibrostenotic disease, several studies could not find this association. Louis et al. found that only disease location and number of flares per year are significantly different between different CD phenotypes, and that ileal disease location was associated with a stricturing disease pattern (30). In addition, although *NOD2* variants were associated with CD susceptibility in a Brazilian population, Baptista et al. could not find a genotype–phenotype correlation (42). The biggest study looking into genotype–phenotype associations in IBD to date, also did not find an association between *NOD2* and fibrotic disease, when considering disease location. They conclude that while disease location is in part genetically determined, it is considered an intrinsic aspect of a patient’s clinical disease and the major driver to changes in disease behavior over time (15).

If there would be an independent association between *NOD2* variants and fibrostenotic disease in CD, how could this be pathophysiologically explained? *NOD2* variants might induce fibrostenotic disease by shifting T lymphocytes toward tissue growth factor beta (TGF-β) cytokine production and by increasing collagen deposition by smooth muscle cells and fibroblasts in the intestine (18). Of the three main variants, functional data are primarily available for Leu1007fsinC: Leu1007fsinC leads to a truncated CARD15 protein, resulting in an altered activation of NF-κB following bacterial triggers (23). Furthermore, it was previously thought that Leu1007fsinC was associated with an impaired IL-1β production and dendritic cell function, resulting in a dysregulation of the antibacterial host defense, increased intestinal permeability, and impaired regulation of innate and adaptive immunity in the intestinal tract (53). However, Maeda et al. later on reported Leu1007fsinC is associated with enhanced NF-κB activation and IL-1β secretion in mice (20). Additional mechanisms, such as diminished mucosal alpha-defensin expression, might also be involved (53). It is possible that the other two variants also alter the structure of LRR domain, resulting in abnormalities in bacterial recognition (35).

### Autophagy-Related 16-Like 1

The *ATG16L1* gene, member of a large family of genes involved in autophagocytosis, is located on chromosome 2q37. It encodes a protein in the autophagosome pathway that is essential in the targeting and destruction of pathogen-derived proteins in the innate immune response (54, 55). Furthermore, autophagy is important for degrading cytoplasmic components, sequestered within vesicles, by the lysosome (21).

After the *ATG16L1* T300A variant (rs2241880) was identified as a susceptibility variant for CD (54, 56, 57), Prescott et al. were the first to associate this variant with ileal disease location, independent of *NOD2* state or disease duration; they did not mention an association with stricturing disease (55). Later on, Fowler et al. reported a significant association between fibrostenotic disease, the GG risk genotype, and ileal disease, independent of *NOD2* (although the number of *NOD2* variants in their Australian CD population might be too small) (58). However, the European IBDchip Project could not confirm this association between *ATG16L1* T300A and fibrostenotic disease (50).

The T300A amino acid substitution is a highly conserved residue that is located in the WD-repeat domain of autophagy-related 16-like 1 (ATG16L1) and which may therefore affect interactions of the protein with other components of the autophagosome (55). This variant plays an important role in pathogen clearance (59), resulting in imbalanced cytokine production (60). Moreover, presence of this *ATG16L1* risk allele seems associated with a reduced ability to generate a specific type of macrophages (Mφind, phenotypically closely resembling the anti-inflammatory CD206^+^ M2-macrophages), also implying an impaired anti-inflammatory functioning (61). The resulting inflammatory signals could eventually stimulate mesenchymal cells to make enormous amounts of collagen and other fibrogenic molecules (62). Importantly, a link between NOD2 and ATG16L1 in the activation of autophagy could also be relevant for intestinal fibrogenesis: it is possible that *NOD2* and/or *ATG16L1* variants jointly can alter the responsiveness of immune cells to bacterial components, thereby amplifying inflammatory signals leading to fibrosis (62). Moreover, the *ATG16L1* T300A variant enhances NOD2-driven cytokine production in an autophagy-independent manner (60, 63).

### Interleukin-23 Receptor

*IL-23R* is found on chromosome 1p31 and encodes a subunit of the receptor for the proinflammatory cytokine interleukin-23 (64). It is highly expressed on the cell membrane of memory T cells and other immune cells, such as natural killer cells, monocytes, and dendritic cells, which identify foreign substances to defend the body against infection. Interleukin-23 receptor (IL-23R) is involved in the mediation of proinflammatory activities by the production of interleukin 17 *via* the activation of Th17 lymphocytes (21).

After Duerr et al. described *IL-23R* as a susceptibility gene to CD (64), Glas et al. published a genotype–phenotype correlation for the rs1004819 SNP within *IL-23R*. This group noticed an increased incidence of ileal involvement and fibrostenotic disease in TT homozygous carriers compared to CC wild-type carriers, which, both however, lost significance after Bonferroni correction (65). We did not find evidence of an association of the main CD-associated SNP in *IL-23R*, rs11202926 (64), with intestinal fibrosis in existing literature.

### Major Histocompatibility Complex

The major histocompatibility complex (MHC) region encodes a large number of immunological proteins, including the antigen-presenting classical human leukocyte antigen (HLA) molecules. Genome-wide association studies of IBD have shown strong evidence of association with the MHC complex (66). Because of the complexity of the region, many researchers avoid including this region into their analysis. One study by Ahmad et al. studied 340 SNPs in 24 genes from the HLA region in relation with fibrotic CD, but did not find an association (19). The IIBDGC genotype–phenotype study found a genome-wide significant association with rs77005575 located in the MHC region and disease behavior, independent of disease location (15). None of the included classical HLA alleles were independently associated with disease behavior in this study.

### Toll-Like Receptors

Toll-like receptors (TLRs) are transmembrane domain protein with a tripartite structure: they contain an extracellular domain (including LRRs) responsible for ligand recognition, a single transmembrane-spanning region, and a globular cytoplasmic Toll/IL-1 receptor (TIR)-signaling domain. Currently, 10 TLRs are described in humans (67). They are expressed in myeloid cells and play a major role both in detecting microbes and in initiating innate immune responses. *TLR4*, expressed in the Golgi apparatus of intestinal epithelial cells (IECs), interacts with LPS, contributing to the perpetuation of inflammatory epithelial cell injury *via* tumor necrosis factor alpha (TNF-α)-induced alterations of enterocyte turnover in an (auto)paracrine matter (38).

In 2004, Franchimont et al. identified rs4986790 (Asp299Gly), within *TLR4* as a susceptibility variant to CD, regardless of association with phenotype (68). This variant is associated with decreased responsiveness to endotoxins in humans (69, 70). Lakatos et al. could not find this association in a Hungarian cohort (possibly because the variant allele is more present in their control population compared to Franchimont et al.), and they also did not find a genotype–phenotype correlation for this SNP (38). Although there is no strong evidence for a role for *TLR4* in the pathogenesis of fibrostenotic disease in CD, Rieder et al. suggested the first direct link between innate immunity to bacteria (*via* TLRs) and fibrosis in humans (71). Furthermore, in other diseases, such as systemic sclerosis and liver fibrosis, *TLR4* might have a pathophysiological contribution (72, 73).

### Fractalkine Receptor 1, CX3CR1

CX3CR1 (previously termed V28) is a leukocyte chemotactic and adhesion receptor that binds fractalkine (CX3CL1 or neurotactin, expressed in epithelial and endothelial cells), a CX3C chemokine that exhibits properties of both traditional chemokines and adhesion molecules (74). CX3CR1 is expressed on natural killer cells, monocytes, CD8^+^, and some CD4^+^ T cells. By binding fractalkine, it regulates the migration of a subpopulation of CD8^+^ intraepithelial lymphocytes into the intestinal lamina propria and their interaction with IECs (74). After stimulation by bacteria (or bacterial degradation products), CX3CR1-expressing cells rapidly adhere to the inflamed vascular endothelium and may play a role as a vascular gateway for cytotoxic effector cells (52).

After two strongly correlated (*D*′ = 0.99) *CX3CR1* polymorphisms (V249I, rs3732379; T280M, rs3732378) were identified in HIV-positive patients (75), Brand et al. investigated these SNPs in the context of CD. They observed an association between both SNPs and fibrostenotic disease (without Bonferroni correction), but this was not independent of ileocolonic disease location (74). Later, Sabate et al. again noticed a trend toward fibrostenotic behavior in V249I carriers (not statistically significant after Bonferroni correction), especially in smokers, independent of *NOD2* Leu1007fsinC carriage and ileal involvement (52). Although the two SNPs are strongly correlated (75), Sabate et al. did not see a similar trend for T280M (52).

Several findings point toward CX3CR1 as a critical component in maintaining homeostasis of lamina propria macrophages, and master regulators of inflammation and fibrosis (76). Importantly, specifically for the described variants, it was shown *in vitro* that peripheral blood mononuclear cells (PBMCs) from individuals with wild-type *CX3CR1* genotype adhere more potently to membrane-bound fractalkine than do PBMCs from homozygous V249I–T280M donors (74, 77). Despite the limited data about an association between *CX3CR1* and fibrostenotic disease, these functional data could point toward a true role for the CX3CR1/fractalkine axis in fibrosis in CD.

### Tissue Growth Factor Beta

Tissue growth factor beta is encoded by a gene on chromosome 19q13. It is a regulatory protein that plays a key role in inflammatory, fibrotic, and immunological events in the intestinal mucosa (78, 79). Enhanced expression of TGF-β and its receptors seems to be involved in the pathogenesis of CD, and might contribute to fibrosis (80, 81). After some SNPs (including C509T) in the *TGF-*β*1*-gene were described to lead to variations in the production of TGF-β serum levels in women (82, 83), some groups looked in vain for an association with susceptibility to CD (79, 81, 84). However, Hume et al. observed a significant association between the AA genotype of a SNP in codon 25 in the *TGF-*β*1* gene and a fibrostenotic phenotype. CD patients homozygous for the profibrotic A allele also tended to have a shorter time to intestinal resection (79).

### Angiotensinogen

Angiotensinogen, mapped to chromosome 1q42, is meant to function locally as a cytokine in several organ systems, participating in the regulation of inflammation and fibrosis. After being cleaved by renin into angiotensin I and processed to angiotensin II, it may increase the production of TGF-β1 (79).

After a gain of function SNP 6 bp from the transcription site was described in the angiotensinogen gene (85), Hume et al. studied its association with CD and CD phenotype. They reported a positive association for the A allele and CD, although without any genotype–phenotype association at the univariate or multivariate level (79).

### Matrix Metalloproteinases and Tissue Inhibitors of MMPs

Matrix metalloproteinases (MMPs), all Zn-activated endoproteinases, are subdivided into four groups, depending on their structure and substrate specificity: collagenases, gelatinases, stromelysins, and membrane-type MMPs (86–88). They mediate degradation of essentially all components of the extracellular matrix. The enzymatic activity of these potentially harmful proteinases is tightly controlled and counterbalanced by endogenous inhibitors, such as alpha 2 macroglobulin, and specific tissue inhibitors of MMPs, the so-called TIMPs. TIMPs are produced by the same cell types that produce MMPs, primarily in cells resembling macrophages and fibroblasts (87, 89).

In the last decade, many different SNPs in these genes were described, related to processes such as fetal development (90), primary sclerosing cholangitis (91), and coronary atherosclerosis (92). Meijer et al. also studied their role in relation to CD susceptibility and CD phenotype. They found that the 5T5T genotype at the *MMP-3* locus (an additional thymidine insertion at −1613 of the *MMP-3* promoter) was associated with fibrostenotic CD (88). Furthermore, expression data of Warnaar et al. demonstrated increased levels of MMP-3 in stenotic and prestenotic resected CD ileum, pointing to an MMP-3 (stromelysin-1)-mediated altered clinical course of CD patients. These findings might explain the high recurrence rate of intestinal strictures, as in non-resected, prestenotic CD ileum in which the anastomosis is made, tissue turnover is present (89). Conflicting evidence exists regarding the consequences of the 5T5T genotype: some groups reported upregulation of MMP-3 expression (93, 94), whereas others reported a downregulation (95). In the study by Meijer et al., patients stratified according to *MMP-3* genotype had similar MMP-3 total activity (88).

### Membrane-Associated Guanylate Kinase, WW and PDZ Domain-Containing 1

*MAGI1* is located on chromosome 3p14 and encodes the membrane-associated guanylate kinase WW and PDZ domain-containing protein 1 (96). This protein plays an important role in the tight junction of IECs through interaction with JAM4, a junctional adhesion transmembrane molecule. Disruption of this epithelial barrier can have dramatic effect on the mucosal integrity, which has been shown to contribute to the development of CD (96).

Alonso et al. recently published an interesting association between fibrostenotic CD and rs11924265, located in a 46.5-kb haplotype block inside a *MAGI1* intron. They validated this association in an independent replication cohort (96). Previously, other groups have shown a significant increase in intestinal permeability in patients with stricturing disease (97). Rs11924265 might induce an alteration in the membrane-associated guanylate kinase, WW and PDZ domain-containing 1 (MAGI1) protein function, contributing to an exaggerated immune response and to the subsequent transmural inflammation of the gastrointestinal tract (96).

### Janus Kinase 2

*JAK2*, located on chromosome 9, encodes for an intracellular tyrosine kinase that transduces cytokine-mediated signals *via* the JAK-STAT pathway (50, 70). The large, retrospective, and multicentre IBDchip study found that rs10758669 (C allele), within the *JAK2* gene, is associated with an increased risk for ileal involvement and stenosing disease behavior. One mechanism by which Janus kinase 2 (JAK2) contributes to this fibrostenotic disease could be by altering intestinal permeability (50). Indeed, Prager et al. previously demonstrated that patients carrying the rs10758669 C risk allele significantly more often had an increased permeability compared with patients without the C allele (98).

### Fucosyltransferase 2

*FUT2*, located on chromosome 19 (70), encodes the secretor enzyme alpha(1,2)-fucosyltransferase (Lewis blood group system), which allows expression of ABO antigens on the gastrointestinal mucosa and in bodily secretions (secretor phenotype) (99). After a nonsense allele in *FUT2*, rs601338 (W143X), was identified as a susceptibility variant for CD (100, 101), Forni et al. found non-secretors to be at slightly higher risk of a stricturing/penetrating behavior (OR 1.51, *p* = 0.046). Additionally, their analysis revealed patients with blood group O are less likely to develop a stricturing disease (OR 0.70, *p* = 0.038) (99). Although it is known that fucosyltransferase 2 (FUT2) expression affects the composition of the gut microbiota (102), the pathophysiological link between this specific SNP and fibrostenotic disease has not been unraveled yet. Theoretically, an altered microbial environment might induce more severe inflammation, leading to a more aggressive phenotype.

### Other Genes

In 2009, Henckaerts et al. examined the influence of some CD-associated susceptibility loci on changes in disease behavior. They found that homozygosity for the rs1363670 G-allele in a gene encoding a hypothetical protein near the *IL12B* gene, located on chromosome 5, was independently associated with stricturing disease behavior, especially in patients with ileal involvement (70, 103). So far, the pathophysiological consequences of this SNP, leading to a non-coding transcript variant, are not fully understood (70).

Because inherited risk factors [factor V Leiden, methylenetetrahydrofolate reductase (*MTHFR*) C677T] have been reported to be associated with fibrosis in other chronic inflammatory diseases, Novacek et al. performed a retrospective study in CD patients aiming to identify these risk factors in fibrostenotic CD. They concluded that the *MTHFR* 6777TT variant, factor V Leiden, and the prothrombin G20210A variant are not associated with fibrostenosis in CD (104).

As TNFα plays a pivotal role in the pathophysiology of IBD, confirmed by the efficacy of anti-TNF drugs, such as infliximab and adalimumab (105), Meijer et al. investigated the association between a SNP (*G308A*) in TNFα and fibrostenotic disease (88). In line with other reports (106, 107), they could not find an association between this SNP and fibrostenotic CD (88).

### The Combined Action of the Known Susceptibility Variants

Crohn’s disease is a complex multigenic disease, where several small-effect risk variants combined influence disease onset. It is more and more suggested that combining the many individually weak signals into a genetic risk score might be a more powerful approach to study the genetic association with subphenotypes or to improve predictive ability of disease (15, 108). Such a genetic risk score was calculated in the IIBDGC genotype–phenotype study and tested for association with several disease subphenotypes. A strong association with disease behavior was found (*p* = 9.23 × 10^−18^), indicating that the known susceptibility loci combined can be a useful measurement of CD subtypes, but still do not have enough predictive ability to distinguish between the different subtypes (15).

## Genetics and Fibrosis in Pediatric CD

Currently, not much is known about the genotype–phenotype association in pediatric CD. Russell et al. studied *NOD2* variants in the Scottish early onset CD population (aged <16 years) and noticed a relatively small contribution to CD susceptibility, but a major impact on phenotype. Presence of stricturing disease behavior at diagnosis showed a trend toward an increase in carriers of *NOD2* variant alleles, which became significant by 2 years of follow-up (39). The association of *NOD2* variants and fibrostenotic pediatric CD was previously already reported by two other groups (109, 110).

In contrast with a study in adult CD (79), Liberek et al. could not find any significant correlation between the four common SNPs in *TGF-*β and any specific clinical parameter (111).

In 2014, Strisciuglio et al. performed a genotype–phenotype correlation study, focusing on autophagy gene variants. They observed a trend toward switching to a fibrostenotic disease in children homozygous for the *ATG16L1* T300A risk allele. They did not find an association between *NOD2* variants and stricturing CD (112).

## Genetics and Fibrosis Around the World

Although the incidence of IBD is rising in developing countries (113, 114), epidemiological data on the clinical phenotype of disease, and genotype–phenotype association studies, in non-European populations are limited. Similar as for Caucasian populations though, several smaller genotype–phenotype studies have been performed in non-Caucasian populations (115–120). These usually study the same variants as those considered in Caucasian populations (*NOD2*, *IL-23R*, etc.), but only one Korean study found the *IL23R* variant rs1004819 associated with stricturing and penetrating disease (119). It is possibly not surprising that *NOD2* variants are not found to be associated with disease (subtypes) in different populations, as *NOD2* variants have been seen with different frequencies in geographically diverse populations. Whereas the prevalence of CD patients who carry at least one *NOD2* susceptibility variant varies from 27 to 50% in most Caucasian European populations, observed frequencies are much lower (15–21%) in Scandinavian countries (121, 122), which are generally characterized by more homogenous study populations. Caucasian populations, relatively far from Europe, but with European ancestry with hardly no racial mixing, such as the United States, Canada, and Australia, have *NOD2* variant frequencies comparable with those found across the rest of Europe (122). In Asians (Japanese, Chinese, and Korean), Arabs, Africans, and African-Americans, the *NOD2* variants are rare or even absent (5, 21, 116).

Recently, the first trans-ancestry association study of IBD was published by the IIBDGC (5). They collected subphenotype data on 1991 patients with CD from East Asia, India, and Iran, and compared these data with available clinical phenotypes for 19,290 Europeans (15). They showed some demographic differences, with, for example, more stricturing behavior and perianal and less inflammatory CD in the non-European population compared to the European population, in line with the previously reported prospectively collected clinical findings in incident cases of IBD in non-Europeans (114). It will be interesting to see if these differences are explained by genetic factors that differ between populations, or rather by environmental factors (including different health-care systems), ascertainment bias, or a combination of these. The trans-ancestry association study showed that although for the majority of the IBD risk loci, the direction and magnitude of effect are consistent in European and non-European cohorts, genetic heterogeneity was seen between divergent populations at several established risk loci, driven by differences in allele frequency (*NOD2*), effect size (*TNFSF15* and *ATG16L1*), or a combination of both (*IL23R* and *IRGM*). A large trans-ancestry genotype–phenotype study is under way, undoubtedly shedding light on possible genetic heterogeneity of disease subphenotypes in different populations.

## The Promise of Epigenetics

So far, the epigenetic control of inflammation and fibrosis in IBD is not fully understood (12). But, as only a modest fraction (ca. 15%) of the 200 association signals is driven by missense mutations, the large majority of causal alleles is likely to be related to regulatory functions such as modulation of gene expression (123). Intestinal disease-associated DNA methylations in IBD do occur, leading to changes in gene expression (124), such as in several loci within the interleukin 12/interleukin 23 pathway (125). As epigenetic changes are dynamically responsive to the environment, they are likely to play a key role in the pathogenesis of fibrosis and offer a molecular explanation for how the intestine becomes profibrotic (126).

Sadler et al. recently published their genome-wide analysis of DNA methylation and gene expression in the context of fibrotic CD (126). They found three functional candidate genes to be differentially methylated and expressed in fibrotic CD: wingless-type mouse mammary tumor virus integration site family member 2B (*WNT2B*), prostacyclin synthase (*PTGIS*), and prostaglandin D2 synthase (*PTGDS*) (126). The decreased expression of hypermethylated *WNT2B* and *PTGIS* are novel findings both in the context of fibrosis and CD, though hypermethylation of the *PTGIS* promoter has been described as a feature of colorectal cancer (127). Interestingly, increased expression of *WNT2B* has been detected in the intestinal mucosa of UC patients (128), suggesting that *WNT2B* may perform distinct functions in CD and UC (126). Hypomethylation of the *PTGDS* gene, together with increased gene expression levels, was previously noticed in a murine UC model (129). In future, both epigenetic and transcriptomic analyses will undoubtedly reveal novel insights in the pathogenesis of stricturing CD, potentially leading to new targeted therapies.

## Clinical Implications

Based on current evidence, it is too early to adjust treatment in CD patients according to genetic profiles, in order to personalize treatment in CD (15). *NOD2* is by far the most studied genetic predictor for fibrostenotic disease in CD, and many studies suggested an important role for *NOD2* variants in developing fibrostenotic CD. Still, the low sensitivity of a single *NOD2* variant for predicting fibrostenotic disease does not justify *NOD2* genotyping in all patients (48), and there is no adequate scientific evidence for a top-down medical therapy based solely on *NOD2* variants. It has been suggested that targeted early-intensive therapy for high-risk patients with two *NOD2* mutations might be beneficial, if proven by prospective trials (47), but so far this evidence does not exist. Importantly, based on the biggest genotype–phenotype study ever done including over 19,000 CD patients, it was found that no *NOD2* variants are associated with fibrostenotic disease after conditioning for disease location. Disease location thus seems to be the major driver to changes in disease behavior over time (15). Preferential involvement of the terminal ileum could be explained by *NOD2* variants abrogating normal Paneth cell behavior, as Paneth cells express NOD2/CARD15 throughout the small intestine, with maximal expression in the terminal ileum (35, 130).

## Conclusion and Future Directions

As outlined above, several genotype–phenotype studies have been performed to find which genetic variants play a role in defining CD disease location and behavior, but hardly any variants were uniformly found as independent risk factors for developing fibrostenotic disease in CD. Different reasons can be put forward. The first one is related to power of the individual studies. Many studies indeed included relatively small patient numbers (Table 1), and looking into subgroups of patients makes the sample sizes even smaller. It should also be noted that various studies might include patient groups from either population-based registries and/or from secondary or tertiary referral centers. This has a direct influence on the proportion of patients with more severe disease as opposed to inflammatory disease, which in turn could lead to over or under presentation of certain genetic associations. An example is the Scandinavian registries that are population-based and where indeed a lower proportion of stenosing and penetrating CD is seen (15). *NOD2* frequencies in these populations are also lower (see above) (121), but this could be linked to the population-based character of the study population. Third, most susceptibility variants are not the pathophysiological causal ones, but are in LD with the true causal variant(s) at that locus, which might have more qualitative or quantitative effects and explain the association with a certain clinical feature. Fourth, many studies apply different definitions for stenosing disease or use a limited number of variables given in the Vienna Classification (41). Another reason could be the dramatic change in disease behavior over the course of the disease, implying disease behavior of CD cannot be analyzed without taking into account the duration of disease (2, 30). Also, because of the importance of disease location in driving changes of disease behavior over time (15), disease location should always be considered when analyzing risk factors for stenosing disease. In the case of, for example, *NOD2*, there is a strong correlation of *NOD2* and ileal disease location (131), which might induce a false, confounded association between *NOD2* variants and fibrostenotic disease in those cases where disease location is not considered in the genotype–phenotype analysis. Finally, disease behavior is influenced by environmental factors (132), which can be dramatically different in the different studies. Examples not only include smoking and NSAIDS use but also specific treatments may hide patients at risk to develop certain subtypes of disease. Any disease behavior and severity analysis should be interpreted with caution, when there is no access to medication use and response to medications, especially for patients in the biologics era.

Among the 163 genome-wide significant IBD susceptibility loci as identified in the study by Jostins et al. (4), genetic variants in immune system components (*NOD2*, *IL-23R*, *IL-12B*, *JAK2*, and *FUT2*) and autophagy [*ATG16L1*, leucine-rich repeat kinase 2 (*LRRK2*)] could (jointly) contribute to the activation of mesenchymal cells and pathogenesis of fibrosis (4, 9, 133). Although these susceptibility genes might pathophysiologically contribute to fibrostenotic processes, not all have been found to be associated with stricturing CD. For example, the *LRRK2* CD-associated M2397 allele inhibits nuclear factor of activated T cells (NFAT) (134), which is known to control fibroblast plasticity in the heart (135). LRRK2 might thus also be involved in fibrosis in the gut, although so far this has not been reported. We did not find any studies where a genetic association was shown between *LRRK2* and fibrostenotic CD. The IIBDGC study by Cleynen et al. in addition found that while many of the known 163 risk loci showed nominal evidence of association with CD behavior, a combined genetic risk score of these individual weak signals was strongly associated with CD disease behavior (15). Still, the development of fibrosis is preceded by a period of initial inflammation, and not all patients with CD express a fibrostenotic phenotype (1, 2, 6). This highlights the possible difference between loci predisposing to overall disease (CD or UC) and loci predisposing to clinical phenotypes (4, 9, 136). It is thus important to consider the idea of different genes driving susceptibility on the one hand and disease behavior on the other. The IIBDGC study for the first time does this on a large scale, but hardly finds any genome-wide significant loci for disease behavior independent from disease location, except from rs77005575 (MHC) (15).

**Figure 1:**
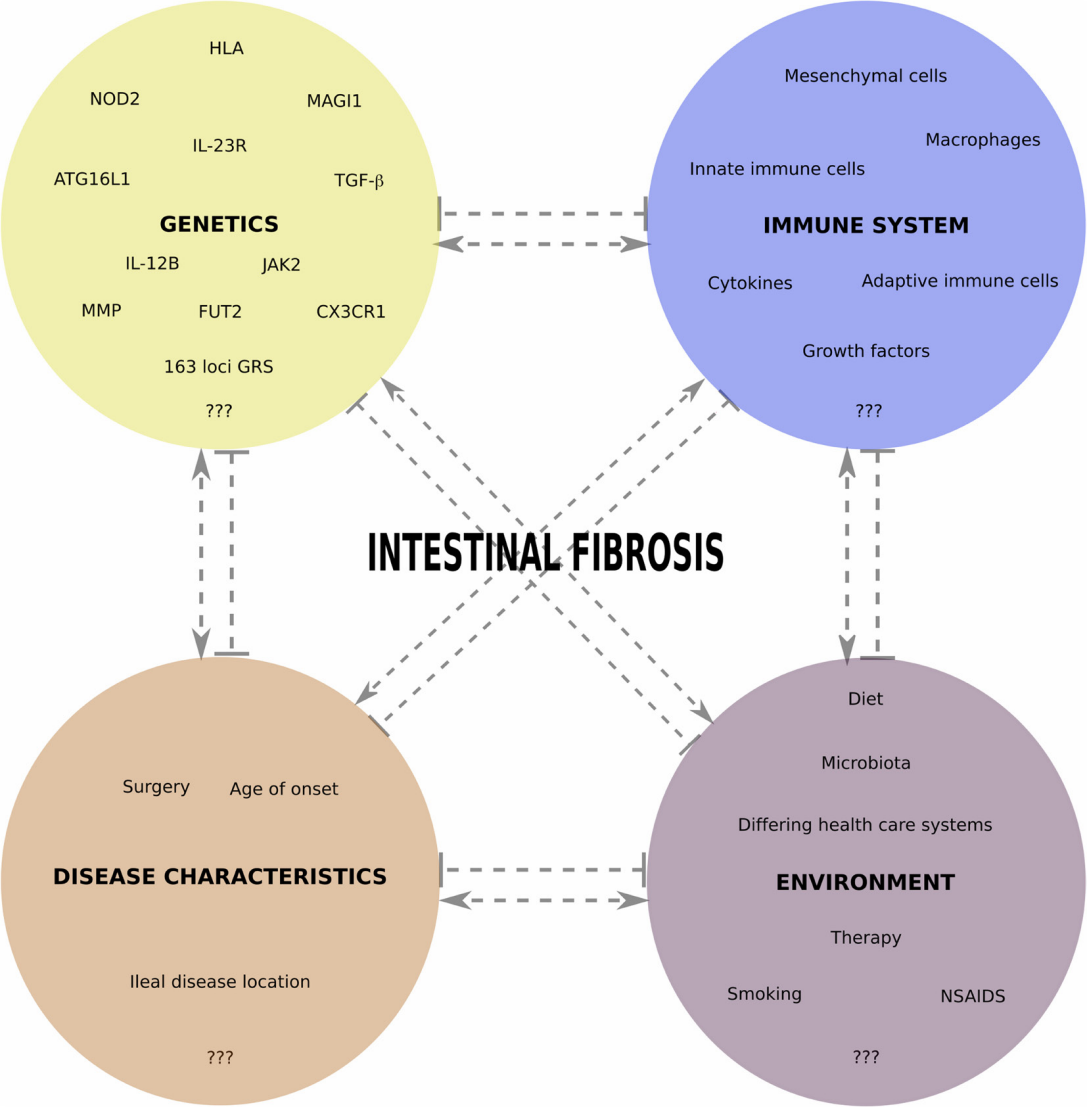
**Overview of different interacting aspects important in fibrotic CD pathogenesis**. In each of the four main categories, we indicate some factors for which a role in fibrotic CD has been shown, with an emphasis on the genetic factors reviewed in this paper. Pointed and barred arrows denote a positive and negative interaction, respectively.

Figure 1 gives an overview of different interacting aspects we believe are important in fibrotic CD pathogenesis and summarizes the genetic factors identified thus far. In future studies aimed at finding the genetic contributions to fibrotic CD, it will be important to consider as much as possible environmental risk factors (smoking, diet, microbiota, medication use, etc.), as well as non-Caucasian populations. To gain additional insights into the pathophysiology of fibrotic CD, and find predictive markers, an integrated analysis, including genetics, epigenetics, transcriptomics, microbiomics, etc., and prospective study setups, will be the next steps.

## Author Contributions

BV: literature search and drafting of the manuscript. IC: drafting of the manuscript and critical revision of the manuscript.

## Conflict of Interest Statement

The authors declare that the research was conducted in the absence of any commercial or financial relationships that could be construed as a potential conflict of interest.
